# “A mismatch between what is happening on the inside and going on, on the outside”: a qualitative study of therapists’ perspectives on student mental health

**DOI:** 10.1186/s13033-021-00508-5

**Published:** 2021-12-20

**Authors:** Maja Anna Marszalek, Helene Faksvåg, Thea Hannestad Frøystadvåg, Ottar Ness, Marius Veseth

**Affiliations:** 1 Department of Clinical Psychology, University of Bergen, Bergen, Norway; 2 Department of Education and Lifelong Learning, Norwegian University of Science and Technology, Trondheim, Norway

**Keywords:** Students, Mental health, Higher education, Qualitative research, Therapist perspective

## Abstract

**Background:**

Although a growing number of studies indicates a high prevalence of reported mental health problems in students and that the problems are growing in number and severity, less is known about the experiences of students facing mental health problems and personnel who interact with students that seek help for these problems. The aim of this study is to examine the perspectives of therapists working with students seeking therapy for mental health problems.

**Methods:**

A qualitative study of 15 therapists’ perspectives. Data are collected with in-depth, semistructured and open-ended interviews, and analyzed using a reflexive thematic analysis.

**Results:**

We developed four themes that summarize how the therapists viewed and understood the students’ experiences of mental health problems: (1) *an agonizing mismatch between the inside and the outside*, (2) *conflicting needs for closeness and distance*, (3) *no room for pain*, and (4) *a major potential for ripple effects*.

**Conclusions:**

We relate our findings to the existing theory and research, and we discuss the implications for clinical practice and the limitations of the study. We argue that our findings demonstrate the need to provide students room to explore and make meaning of their difficulties and pain related to mental health problems, in addition to offer some symptom-reducing tools and techniques that can enhance performance and academic achievements. More research is needed to understand what student mental health problems might be related to and what students need.

## Background

In recent decades, concern has been increasing about student mental health [1–4]. Studies suggest a high prevalence of reported mental health problems among students [1, 4–7] and that these problems have grown in both number and severity [2, 8]. Moreover, studies indicate an increase in students seeking help for mental health problems [9] and that this increase has contributed to the perceived pressure on the capacity for the mental health services offered by college counselling and mental health centres (CCMHC) in US institutions [10].

Although many studies indicate that mental health problems are highly prevalent among students, it remains unclear how the numbers can be understood. Some question whether the occurrence of mental health problems has increased or whether the numbers reflect an increase in help-seeking behavior [9]. Some relate the occurrence of mental health problems among students to their age, arguing that it is associated with an increased risk for mental health problems [3] and to the changes and challenges students face, such as identity exploration and development [11, 12]. Others have looked more specifically at the characteristics of being a student that may be associated with mental health problems, e.g., increased stress related to debt and financial concerns [13].

The database of studies on student mental health is growing, including knowledge on the prevalence and severity of mental health problems and possible factors associated with students’ mental health. However, less is known about the experiences of students facing these problems and the perspectives of personnel who interact with these students in different settings. One study interviewed college students about their mental health attitudes and found that the students perceived stigma as a barrier to treatment seeking for mental health problems, felt that an increasing education and awareness and regarded compassion and understanding to those experiencing mental health problems as important for reducing stigma-related attitudes [14]. Some studies have explored the perspective of people who meet and work with students in different ways, e.g., how clinicians in campus mental health centers experience and respond to managerial practices [15]. Based on interviews with university staff, McAllister et al. [16] found that the staff often interacted with students experiencing mental health problems and played an important role in supporting these students. When interviewing counselors about changing services due to economic pressure, the findings showed that they experienced that the changes resulted in more efficient services that seemed to be more acceptable to the students and increased the workload of the counselors [17]. In line with the quantitative studies mentioned earlier, administrators of CCMHCs, experienced that the severity of mental health concerns and demand for student mental health services had increased for the past years [10]. They related the increased severity of concerns in the student population to psychosocial differences compared to prior generations [10]. Some reflected upon how societal pressures may have contributed to increased anxiety among students, and that students are more competitive in order to be successful. Others discussed how students of today have grown up being more dependent on their parents and technology, leaving them with fewer opportunities to develop coping skills and ability to tolerate stress on their own [10].

To summarize, the main focus of the qualitative studies exploring the perspective of university staff and clinicians seems to be the support, and counselling and treatment services provided, rather than the students and the mental health problems they experience. To our knowledge, few studies have explored therapists’ views on students’ mental health. Therapists in counselling and mental health centers interact with students by providing counselling and treatment for students seeking help for mental health problems. These therapists have extensive knowledge and training related to mental health and processes that are to help people make changes that can prevent and treat mental health problems, and promote mental health in general. Based on the combination of possessing expertise on mental health and interacting with students that are seeking help and support because of mental health problems, we argue that therapists’ first-hand experiences provide valuable insight into student mental health. Student mental health is associated with wellbeing, academic success and retention (e.g., [1, 4]). Adding knowledge to the database about student mental health from the perspective of therapists can benefit efforts to develop interventions that prevent and treat mental health problems among students and support them so they complete their studies and transition into the workforce.

In the present study, we aim to gain insight into and develop knowledge about student mental health through an in-depth exploration of the following research questions: *How do therapists view and describe students seeking therapy for mental health problems? How do they understand students’ experiences of mental health problems?*

## Methods

### Study setting

In Norway, all citizens are ensured public health care services [18]. For students, counselling and mental health care are offered free of charge and without the need for a referral. Typically, these services are provided by nonprofit student welfare organizations who offer short-term psychotherapy, group therapy and a variety of courses on campus and/or digitally. These services are interdisciplinary, and student welfare organizations also provide a long range of other services, including career counselling, housing, child care, general medical services, and sports- and leisure-activities.

### Design

This article is based on a qualitative project that explored the lived experiences of therapists working with students seeking therapy. For this purpose, we applied a reflexive thematic analysis [19] to identify patterned meaning across data obtained from in-depth, semistructured and open-ended interviews [20]. This approach allowed us to create and represent important aspects of the therapists’ subjective experiences working with student mental health.

### Reflexivity

At the time of the data collection and initial steps of analysis, the first, second and third authors were students and practicing psychotherapy. Currently, the first, second, third and fifth authors are clinical psychologists, and the fourth is a family therapist and counselor. The fourth author is a professor, and the fifth author is an associate professor. All authors have an interest in community mental health and humanistic, relational and psychodynamic approaches to the field of psychotherapy. We share an interest in qualitative research on mental health.

### Data collection

The first, second and third authors conducted 15 individual in-depth interviews. The participants in this study were recruited using a convenience [21] and a snowball-sampling technique [22]. First, we contacted leaders of centers of the student welfare organizations that offered mental health services from the eastern and western parts of Norway and invited them and their colleagues to participate. In addition, we asked acquaintances with connections to possible participants and the interviewed therapists about contact information for therapists that might have relevant experience and interest in participating in the study. Of the fifteen participants, eleven were women and four were men. We considered this an adequate sample size to obtain meaningful units of information from the data [23].

We developed a semistructured interview guide [20] to facilitate a flexible and exploratory dialogue with the participants. See Table 1 for the main themes in the interview guide. In the invitation to the project, the participants were asked to think of a specific case in which the student expressed that the therapy had been of help to them. The interviews were conducted at the participants’ workplaces or a location of their choice between April and August 2019. After the interview, they were verbally asked about demographic information or to fill out a written form. The interviews ranged from 51 to 109 min. All interviews were audio-recorded and transcribed by the first three authors. Each author transcribed the interviews that they conducted.

**Table 1:** Main themes in the interview guide

What did you and the student experience as the student’s problem?
How did you and the student work with this problem in therapy?
What do you consider the student gained from the therapy?
How did you perceive the impact of external factors and the student’s attributes on the student’s mental health?
How do you perceive student mental health today, compared with when you were a student?
How do you experience the impact of the way we talk about mental health problems on student mental health?
What do you consider the students need to have a good mental health?

### Participants

The participants’ ages ranged from 30 to 62 years, with a mean age of 45 years. Eleven were licensed clinical psychologists, one had a master’s degree in psychology, one had basic education as a nurse, and one was educated as a social worker. The years of experience working with student mental health varied from 9 months to 25 years. Within the last 5 years, they were employed by the student health services in the western, eastern and southern parts of Norway while working with students. Most were still working with students as their clients when the interviews were conducted, either at student health services or at private clinics. The therapists reported different theoretical orientations and advanced trainings, including emotion-focused therapy, sensorimotor psychotherapy, psychodynamic psychotherapy, mentalization-based treatment, systemic therapy, humanistic and existential approaches, mindfulness-oriented approaches, solution-focused approaches, cognitive therapy and motivational interviewing.

### Data analysis

We used a team-based [24] and reflexive approach to the thematic analysis [19] to describe common, recurring patterns across the interviews. Our aim was to create themes, which, according to Braun and Clarke [25], refers to constructs that “capture something important about the data in relation to the research question” (p. 82). We reflected upon and discussed how our preconceptions could influence the different steps of this approach throughout the study. We followed the following six steps [19, 25].

1. We read through all the data to obtain a sense of the descriptions and recurring patterns across them.
2. We examined the parts of the interviews that were related to the research question and registered units of meaning that represented different aspects of the therapists’ descriptions and understandings.
3. We explored the units of meaning, and, based on broader patterns of meaning across the units, we searched for tentative themes.
4. We examined the tentative themes and made sure they answered the research question and described central aspects of the therapists’ descriptions.
5. We defined the contents of and gave names to each theme, that will be described in the findings section. 
6. All authors contributed to writing the final report.

### Ethical considerations

This project is not subject to Norway’s Health Research Law, as we only explored the therapists’ views and not those of the clients. Therefore, approval from The Regional Committee for Medical and Health Research Ethics was not applicable. The Norwegian Center approved the study protocol for Research Data (Project Number: 384543) prior to conducting the interviews. The participants received written and verbal information about the objectives of the study and were informed that participation was voluntary and that they could withdraw from the project without giving a reason at any time.

## Results

We organized the therapists’ descriptions into four themes: (1) an agonizing mismatch between the inside and the outside, (2) conflicting needs for closeness and distance, (3) no room for pain, and (4) a major potential for ripple effects.

### An agonizing mismatch between the inside and the outside

An incongruence emerged across the interviews between the students’ inner lives of suffering and despair and outer appearances. Several therapists described that, even though the students had mental health problems to different extents and severities, they usually functioned well and had stabilized many aspects of their lives. They had jobs, social networks, stable economic situations and fixed living situations. In one of the interviews, the participant characterized this contrast as “a mismatch between what is happening on the inside and what is going on on the outside”. Another therapist stated that it was important to not be blinded by their successful facade, thinking everything was okay based on the students’ appearances. She further elaborated that, when the gap was larger between how the students appeared on the outside and how they felt on the inside, they had more shame and greater difficulty putting it into words. Furthermore, a third therapist emphasized how the students were able to cope, despite suffering as illustrated by the following quotation:

They just want to get a grip on their lives. Additionally, despite very severe symptoms that would cause others not to function, they sometimes miraculously manage to keep … to keep their life going even though they are in processes that are truly burdensome, and as they have stories from hell, truly, even though they are truly traumatized. So there is much serious stuff, but they just want to get a grip on their lives. So it is a wonderfully inspiring group to work with, I have to say.

The descriptions implied that the students’ tendency to criticize themselves facilitated this discrepancy. Most of the therapists underscored how the students had inner critics that caused them shame and emotional pain. However, self-criticism was also described as helpful to the students for attaining success in different areas because it pushed them to give everything they had. One therapist illustrated the duality of self-criticism by characterizing it as a “double-edged sword”, and another explained how it worked as both “an enemy and an asset”. A third participant clarified the mixed consequences of self-criticism in the following way:

Participant (P): That is, this enormous self-criticism. And kind of, contempt for [one’s] own weakness, that was *enormously* prominent. And the paradox with self-criticism is that it is very useful at the same time as it is harmful because it is in a way a driving force, a driving force to get things done, to, kind of, survive and do things right, like she did, right. So that … that is what drives this superficial, apparent coping. So this is what maintains her level of functioning very much. This enormous ….

Interviewer (I): Self-criticism ….

P: Yes, this kind of ruthlessness towards herself. So, with regards to mental health, it is a catastrophe, but with regards to life it is … it works. It is functional. So that was probably a trait that caused her to have a hard time with herself.

These statements illustrate how the therapists viewed the students’ inner critics as contributing to pain and as something that motivated the students to get things done and succeed.

### No room for pain

When we asked the therapists about how they perceived the students’ problems, several spoke about the students having difficulties tolerating and accepting painful thoughts and feelings. A repetitive pattern that occurred across the descriptions was the students perceiving themselves as weak for having these thoughts and feelings and responding to them with merciless self-criticism and self-loathing. This pattern was illustrated by descriptions of the students saying they were “lame” or “stupid” when outlining their problems. The therapists explained how the students appeared ashamed of themselves and beat themselves down with accusations of how they should be, think, feel and act differently than they did.

The participants emphasized the need to create space where the students could share the parts of themselves that they perceived as weak and be with the pain and try to understand whatever was causing it without feeling shame or being self-critical. One therapist explained that it was essential to let the student feel whatever she was feeling:

(…) focused on that it was okay to, with self-acceptance, then, and focused on that she ought to think it was difficult, and […] was allowed to say that it was tough for her, and I was concerned with meeting her that this [experience] seemed tough, and this was much the project. And that she was going to practice, in a way tolerate, experiencing that it was exhausting, because all her life until then she had worked a great deal with, yes. ”However, I don’t have a choice, so I just have to tolerate this”. And then she started to see that, yes, “Now I don’t tolerate this any longer, and then it is something wrong with me”. So, she felt weak and that she should have tolerated it in a way because if she does not tolerate it, everything breaks down.

Another participant believed that, compared to previous generations, the students now have more trouble accepting and showing all parts of themselves. In the interview, he said:

It seems that people are … They are … I would not say weaker, but they are more fragile, […] they have less tolerance for their own weakness. That is, they have less room, both for themselves but also for others, in regard to showing that one is … that things are not right.

Across the therapists’ stories occurred the commonality of an apparent tension between the students’ yearning for a “quick fix” to relieve the pain and the therapists’ belief in the need to go deeper to explore and try to understand what was causing pain to facilitate positive change. Several participants described that the students typically asked for techniques and tools that could eliminate the symptoms they were experiencing. This type of focus also became evident in the students having ideas that they would feel better if they learned how to think more positively and realize that their lives were good or if they got a grip and stopped having negative thoughts and feelings. One of the participants reflected upon how these requests for techniques and tools could be a manifestation of the students being accustomed to controlling things, being solution-focused and concrete and eliminating difficult thoughts and feelings instead of trying to tolerate them or understand their meaning.

With regard to this theme, some therapists expressed a concern that using diagnostic terms, such as “social phobia” and “depression”, to label the students’ problems could have a negative impact by reinforcing the perceptions of something being wrong with them and them having problems that needed to be fixed and removed.

### Conflicting needs for closeness and distance

The students appeared to long for closeness while wanting to keep others at a distance, according to the therapists’ accounts. “An extreme number of them say they miss close relationships” a therapist said, and several therapists talked about how the students often expressed that they felt alone with their problems and missed having someone in their lives with whom they could be open and sincere. However, the students often pulled back from others, thus contributing to the distance. The students’ need for connection seemed to be blocked by their need to protect themselves, which they did by keeping others at a safe distance. Furthermore, this dynamic was understood by the therapists as contributing to the students’ loneliness and feelings of being isolated.

Some of the participants related the constraining fears of being perceived as “weak”, “a burden”, “different”, and “something being wrong with them” to the students having an ideal of making it on their own. Some emphasized that it had to do with experiences of not being understood or taken care of when they were distressed. Other therapists related these fears to stories in which the students had been bullied or left out by peers. In the following, a therapist expressed the way the students struggled to feel safe by keeping relationships superficial, even though they wanted close relationships:

Yes, or that can be very typical, that I see many of the students also have […] they have problems trusting other people. Struggling to form close relationships with other people, and they have difficulties with trust, difficulties trusting people, don’t want to let them in completely, keeping them, in a way, at arm’s length. But [they] still suffer because they really want to be close to these people. They want a closer relationship. But then they are contributing to keeping the person away because they are afraid of being let down or afraid of being rejected and hurt ….

The therapists exemplified how the students actively maintained a distance by not putting themselves in vulnerable positions in different ways. Several therapists told stories in which it appeared that the students tried to not reveal that they were having problems, thus hiding the parts of them that could be perceived as “weak”, “different” or “a burden”. One therapist described how a student intentionally had serious conversations about problems with friends at a café to avoid going too deeply into them. Another therapist illustrated this theme with a story about a student that continued to exclude herself from the student fellowship and thus repeated past experiences of being left out. Moreover, one therapist reflected upon how the students’ use of diagnostic labels when talking about problems reflected a low degree of openness and vulnerability, as it shifted the focus away from the painful experiences she had. One participant shared an understanding that the students found it less scary to announce that they had “social anxiety” than to say they were ashamed of themselves and were thinking that others looked at them as “weak and ugly”.

The students appeared to maintain a distance in an effort to save themselves from becoming hurt. However, doing so seemed to have the paradoxical effect of making the students feel lonelier and more different from others, which caused more suffering.

### A major potential for ripple effects

A recurrent theme that emerged from the interviews was that relatively small steps in therapy could have a huge impact on the students’ health and relationships. While the students were suffering, they had a large number of personal resources that held great potential for change and growth. For instance, the participants emphasized how the students often took responsibility for their own health and lives and that they were motivated and willing to take part in a reflective process to understand themselves better. Several participants noted their immense internal drive. The students often had a positive attitude signaling “help me understand what this is about, and then I’ll probably manage”, one therapist stated.

Specifically, the therapists experienced that a new and meaningful experience for many of the students was opening up and being accepted when they revealed experiences of being in pain and ashamed of parts of themselves. This experience appeared to inspire the students to open up about their issues and show their whole selves to family and friends. Consequently, the students felt closer to their families and friends. This phenomenon was demonstrated in the story about the student who was afraid that his story of being left out and losing friends would repeat itself if he opened up about how he was actually doing:

P: No, if he is open about not doing well and says that he has many worrying thoughts and that he is not as happy as he pretends to be, right, he is more himself then. A little more for better or worse. But, so, and it is clear now that he’s in a phase where he can try this out and notices that he can be slightly more himself, that he can discuss and talk about how, and he also says “When I am more open, it turns out that other people also have some problems”, so he kind of gets ….

I: He is experiencing that he, in a way, is more genuine and that other people also open up.

P: Yes, for him then.

I: How has he, how has it been for him?

P: I’ve understood that it has only been positive. That he, kind of, can be a good friend or, yeah, that it goes both ways then.

I: That he kind of, I think I remember you said a moment ago that the relationships become ….

P: Mhm, stronger.

The excerpt illustrates how the student gradually obtained the courage to open up to people outside therapy, which seemed to facilitate authentic and reciprocal relationships.

## Discussion

This study explored and presented an analysis of the accounts of 15 therapists working with students seeking help for mental health problems. An overarching finding was accounts of incongruence between the students inside experiences and outside appearance, duality of coping strategies and the perception of innate forces in students that helped them cope and make changes, even though they experienced mental health problems. Different aspects of the therapists’ perspectives on student mental health were summarized in the following four themes: (1) an agonizing mismatch between the inside and outside, (2) no room for pain, (3) conflicting needs for closeness and distance, and (4) a major potential for ripple effects.

As illustrated in “no room for the pain”, the participants experienced that the students had difficulties coping with painful experiences and communicated that they wanted the therapists to help them get rid of the experiences. This request can be related to literature on the medical model of psychotherapy [26]. A person’s thoughts, feelings and actions influence and are influenced by the sociocultural ideas, practices, institutions, products, artifacts, and economic and ecological factors that comprise the culture [27]. The medical model of psychotherapy is suggested to be a dominant framework for understanding and treating mental health issues [26]. Following Markus and Kitayama [27], we may argue that this model has an impact on how discomfort and mental health problems are perceived and related to, as demonstrated by the descriptions of the therapists. The students’ ambition for eradicating negative thoughts and feelings, and asking for specific techniques and tools from the therapist to accomplish this, resonates with this model. This is because this view of treatment of mental health problems involves a understanding of why the individual is having the problem and applying specific interventions according to “expert” knowledge, such as offering psychological tools that can `fix´ mental health problems [26]. This also resonates with Zolas [28] descriptions of the influence of medicine on society in that it is making people think something is organically wrong with them and that much can be done medically to make them feel, look or function better. Similarily, the therapists described the students expressing that they felt something was wrong with them and that they needed some psychological tools and techniques to get this fixed so that they could feel and function better. Furthermore, the students’ requests for pain-relieving techniques fit with observations of a modern society, making insistent attempts to eliminate and prevent pain, as it is regarded as something exclusively negative that should not occur [29]. Following Vetlesen [29], we ask: How are students supposed to feel that there is room to experience negative feelings and pain when they live in a culture that fears discomfort, including pain, which is an unavoidable and essential part of human life? Based on our culture’s way of relating to pain in combination with a dominant framework for psychotherapy that focuses on symptom-relieving interventions, we argue that an illusion is constructed that life can be lived without symptoms and pain.

Furthermore, the participants’ descriptions of the students using diagnoses for mental disorders to talk about their problems, as illustrated in our themes “no room for pain” and “conflicting needs for closeness and distance”, resonates with the disease model for mental health problems, which has been criticized for pathologizing human experiences [30]. In these themes, several participants appeared to have similar concerns, as they recounted the negative consequences of using diagnostic terms to talk about their difficulties, such as contributing to experiences that there was something wrong with them that needed to be fixed and being a way of diverting attention away from what they experienced as painful. Szasz [31] warned about using labels such as “mental illness” for what he argued are “problems in living” and not a disease. He claimed that it creates an idea that mental illness is a disease one can *catch, get, have* or *harbor* and finally get *rid* of. According to Binder [32], a consequence of the tendency to describe mental pain and discomfort in terms of diagnostic categories is that we lack a language for the fact that things can never perfect. However, this does not mean that experiences of pain and discomfort are a manifestation of illnesses; instead, they are an essential part of life. He claims that a vocabulary is needed to understand the mundane struggles and challenges in life, where mental pain will always become intolerable at some point and where suffering will always be a part of life—as well as happiness, mastery and companionship [32].

The students’ request for tools and techniques can be understood as a wish to acquire coping mechanisms similar to those they were already using. That is, they want to avoid the parts of themselves that were in pain and which they were ashamed of, as illustrated in “no room for pain”, and they do not want to expose these parts to others, as described in “conflicting needs for closeness and distance”. This resonates with Goffmans [33] understanding of stigma, as it seems that the suffering and vulnerable parts of students are incompatible with the identity as a mastering and successful student, and that exposing these parts might pose a risk of being discredited and be a barrier for doing so. The negative aspect of using such strategies may be that they require the students’ to distance themselves from essential aspects of being human, and therefore what unites us—leaving them more isolated. Accordingly, they risk losing the potential for growth and personal development by getting to know themselves and identifying what matters to them. Binder [32] argues that we need to actively relate to and be able to handle the anxiety and painful feelings that are evoked in us but that we sometimes need to set it aside for a while and address it later, for example, if we experience a loss immediately before an important deadline. Our findings can be understood as an indication that never ending pressure to perform and maintain a façade prevents students from ever readdressing problems associated with anxiety and pain.

Self-criticism was an additional coping mechanism commonly observed by our participants, as highlighted in the theme “an agonizing mismatch between the inside and the outside”. One participant characterized self-criticism as a “double-edged sword”, and several of the therapists regarded it as both a major contributor to the pain and shame the students experienced and a facilitator of the success they achieved. Growing literature has demonstrated the flip side of self-criticism; studies suggest that it is associated with psychological suffering and plays a role in different mental health problems, including depression [34–36], depression in borderline personality disorder [37], posttraumatic stress [38, 39], eating disorders [40], social anxiety [41] and suicidality [42, 43]. The relation between self-criticism and psychopathology has also been demonstrated in the extensive literature on perfectionism. Self-criticism can be regarded as a dimension of perfectionism, as perfectionism has been proposed to involve the combination of high standards of performance and overly critical self-evaluations of one’s own behavior [44]. Similar to studies on self-criticism, findings suggest an association between perfectionism and psychological distress in students [45].

In light of the studies that relate self-criticism to psychological suffering and maladjustment, it may be hard to understand its occurrence among students. However, as described by our participants, self-criticism could also be regarded as a resource for the students. Gilbert et al. [46] emphasized the importance of looking at the function of self-criticism. In our findings, it seemed to serve the purpose of correcting and improving the self of the students they described [46]. The function of self-criticism as a strategy for improvement corresponds with descriptions of self-critical individuals being achievement-oriented and competitive [47].

Based on our discussion, we might assume that self-criticism can be especially beneficial in a setting in which academic performance is valued and the level of competition is high. Furthermore, this resonates with a recurrent rationale for investments in student mental health: enhance students’ competence and academic achievements, increase retention, and prepare students to become the future workforce [1, 4]. These arguments seem to convey a message that students’ tasks are centered on performance and becoming a resource for society. In a study examining the increase in perfectionism in college students over time in light of cultural changes, it was suggested that students might have adopted perfection as a way of coping to feel safe, connected and worthy in a culture with competitive individualism [48]. Following Curran and Hill [48], we argue that students’ efforts to succeed and maintain a mastering façade—as illustrated in our theme “an agonizing mismatch between the inside and outside”, including the use of self-criticism—can be regarded as strategies to secure approval from others.

The same academic culture that seems to reward strategies that enhance performance and achievements, such as self-criticism, paradoxically also leaves little room to slow down and address the painful experiences that may result from using such strategies.

### Implications for practice

It might be tempting to focus on offering problem-fixing tools and techniques, in accordance with the students’ requests, so they can optimize their potential as a future workforce. However, the findings in this study suggest that this group of students can benefit from being offered a room where they feel supported in exploring and giving meaning to the difficulties and pain they are experiencing [29], in addition to focusing on acquiring additional skills that can reduce symptoms and enhance functioning. By expanding their understanding, students with mental health problems may find ways to accept and handle their difficulties and pain. In some cases, they may come to the realization that they do not have an illness or that something is wrong with them psychologically, and that to find a way to live with problems and pain in life does not necessarily mean they need psychotherapy. By experiencing that they are accepted and understood by therapists when facing hardships, they might accept themselves more and learn that it can be safe to show all parts of themselves and be vulnerable in relationships outside therapy. Furthermore, students can benefit from a shift in the way we as a culture and society talk about them as more than the future workforce. They may also profit from talking about pain as a natural part of living that is to be accepted and explored, with the potential for growth [29, 32].

### Reflexivity

In accordance with a reflexive thematic analysis [19, 25], we have tried to be self-aware and transparent about how our roles, perspectives and experiences may have influenced our work throughout all stages of the study [19]. The first three authors conducted the interviews and performed most of the analysis while training as students in the role of therapists. Standing in this gap, with one foot in the students’ world and one in the therapists’ world, we may have been able to relate to and understand the therapists’ accounts of the students in a way that we assume would be true for both the students and therapists. However, this double role may also have caused distortion, as it may have contributed to more focus on some aspects at the cost of others, overlooking potential directions and themes that may be important for obtaining a better understanding of student mental health. To balance this potential, the double role was discussed and reflected upon throughout the study individually and with the fourth and fifth authors, who are at different points in their careers.

Another possibility is that the focus on mastery and achievement may have been influenced by the fact that the first three authors were students and novice therapists, interviewing therapists about students. Both students and therapists might represent groups that are more occupied with academic success compared to the general population, which may have contributed to recognizing this as a prominent theme. Moreover, it may have influenced the interactions and contributed to either party wanting to appear competent and masterful and expecting the other party to appear the same.

### Limitations

Our results are contextually dependent on the participating therapists and their unique experiences with students. Therefore, the findings have limited transfer value and must be interpreted with caution. The number of participants in this study was limited and not randomly selected. Participation demanded spending time and being exposed, and this may have influenced the decision to participate. If the interviews were done by other researchers and with other therapists, students or relatives, the study might have yielded different results than in the current one. Moreover, they were asked to think of specific cases with students who expressed that the conversations were helpful, and they based their experiences on meetings with students seeking help for mental health problems. We assume that the results would be different if we requested experiences related to students who did not express that the conversations were beneficial or who experienced them as making the problems worse or if we asked questions about students in general. However, by asking about specific cases, the participants were given the opportunity to go into detail and explore their perspectives in depth.

Another limitation of this study was the lack of differentiation between mental health problems that could be regarded as severe or moderate. The participants reported diversity with regard to the type and severity of mental health problems. In the process of identifying patterns of meaning that recurred across the therapists’ descriptions, we might have overlooked significant information with regard to variations in mental health problems. However, we offered an open starting point for the interviews, allowing the participants to make decisions based on what they regarded as significant.

## Conclusions

In an effort to understand the perspectives of people witnessing student mental health issues close up, we explored 15 therapists’ experiences with students seeking help for mental health problems. Based on their views and understanding of student mental health, we developed the following themes: (1) *an agonizing mismatch between the inside and the outside*, (2) *conflicting needs for closeness and distance*, (3) *no room for pain*, and (4) *a major potential for ripple effects*. Our findings underscore the need to offer students room to explore and give meaning to their difficulties and pain related to mental health, in addition to offering symptom-reducing tools and techniques that can enhance performance and academic achievements.

More research is needed to understand what student mental health problems might be related to and what students experiencing mental health problems need. Future studies should explore how students experience and understand their mental health issues. Moreover, by exploring the perspectives of the closest relatives of students with mental health problems, we may obtain a better understanding of students’ mental health. Furthermore, the impact of individual, relational, and cultural and societal factors, and their interplay on student mental health should be further researched.

## Data Availability

The datasets for this study are not readily available because they consist of interview data, for which confidentiality cannot be safeguarded. Therefore, the data will not be made available. Requests to access the datasets should be directed to MV, marius.veseth@uib.no.

## Abbreviation

CCMHC: College counselling and mental health center

## References

[1] Evans TM, Bira L, Gastelum JB, Weiss LT, Vanderford NL (2018). Evidence for a mental health crisis in graduate education. Nat Biotechnol.

[2] Knapstad M, Heradstveit O, Sivertsen B. Studentenes helse-og trivselsundersøkelse 2018. https://shotstorage.blob.core.windows.net/shotcontainer/SHOT2018.pdf. Accessed 21 Nov 2020.

[3] Macaskill A (2012). The mental health of university students in the United Kingdom. Br J Guid Couns.

[4] Stallman HM (2010). Psychological distress in university students: a comparison with general population data. Aust Psychol.

[5] Haile YG, Alemu SM, Habtewold TD (2017). Common mental disorder and its association with academic performance among Debre Berhan University students, Ethiopia. Int J Ment Health Syst.

[6] Othman N, Ahmad F, El Morr C, Ritvo P (2019). Perceived impact of contextual determinants on depression, anxiety and stress: a survey with university students. Int J Ment Health Syst.

[7] Zivin K, Eisenberg D, Gollust SE, Golberstein E (2009). Persistence of mental health problems and needs in a college student population. J Affect Disord.

[8] Twenge JM, Gentile B, DeWall CN, Ma D, Lacefield K, Schurtz DR (2010). Birth cohort increases in psychopathology among young Americans, 1938–2007: a cross-temporal meta-analysis of the MMPI. Clin Psychol Rev.

[9] Hunt J, Eisenberg D (2010). Mental health problems and help-seeking behavior among college students. J Adolesc Health.

[10] Watkins DC, Hunt JB, Eisenberg D (2011). Increased demand for mental health services on college campuses: perspectives from administrators. Qual Soc Work.

[11] Arnett JJ (2000). Emerging adulthood. A theory of development from the late teens through the twenties. Am Psychol.

[12] Binder PE (2018). Hvem er jeg. Om å finne og skape identitet.

[13] Jessop DC, Herberts C, Solomon L (2005). The impact of financial circumstances on student health. Br J Health Psychol.

[14] Vidourek RA, Burbage M (2019). Positive mental health and mental health stigma: a qualitative study assessing student attitudes. Ment Health Prev.

[15] Jodoin E, Ayers D (2013). Professional norms versus managerialism in campus mental health centers: the experiences of eight clinicians. J Stud Aff Res Pract.

[16] McAllister M, Wynaden D, Happell B, Flynn T, Walters V, Duggan R (2014). Staff experiences of providing support to students who are managing mental health challenges: a qualitative study from two Australian universities. Adv Ment Health.

[17] Randall EM, Bewick BM (2016). Exploration of counsellors’ perceptions of the redesigned service pathways: a qualitative study of a UK university student counselling service. Br J Guid Couns.

[18] Veseth M, Stige SH, Binder PE (2019). Medicine and meaning—how experienced therapists describe the role of medication in recovery processes in bipolar disorder. Couns Psychother Res.

[19] Braun V, Clarke V (2019). Reflecting on reflexive thematic analysis. Qual Res Sport Exerc Health.

[20] Brinkmann S, Kvale S (2015). InterViews: learning the craft of qualitative research interviewing.

[21] Saumure K, Given L, Given LM (2008). Convenience sample. The SAGE encyclopedia of qualitative research methods.

[22] Noy C (2008). Sampling knowledge: the hermeneutics of snowball sampling in qualitative research. Int J Soc Res Methodol.

[23] Malterud K, Siersma VD, Guassora AD (2016). Sample size in qualitative interview studies: guided by information power. Qual Health Res.

[24] Binder PE, Holgersen H, Moltu C (2012). Staying close and reflexive: an explorative and reflexive approach to qualitative research on psychotherapy. Nord Psychol.

[25] Braun V, Clarke V (2006). Using thematic analysis in psychology. Qual Res Psychol.

[26] Wampold BE, Imel ZE (2015). Counseling and psychotherapy. The great psychotherapy debate: the evidence for what makes psychotherapy work.

[27] Markus HR, Kitayama S (2010). Cultures and selves: a cycle of mutual constitution. Perspect Psychol Sci.

[28] Zola IK (1972). Medicine as an institution of social control. Sociol Rev.

[29] Vetlesen A (2009). A philosophy of pain.

[30] Kinderman P (2014). A prescription for psychiatry.

[31] Szasz TS (1960). The myth of mental illness. Am Psychol.

[32] Binder PE (2020). En kort introduksjon til eksistensiell psykologi.

[33] Goffman E (1963). Stigma: notes on the management of spoiled identity.

[34] McIntyre R, Smith P, Rimes KA (2018). The role of self-criticism in common mental health difficulties in students: a systematic review of prospective studies. Ment Health Prev.

[35] Ehret AM, Joormann J, Berking M (2015). Examining risk and resilience factors for depression: the role of self-criticism and self-compassion. Cogn Emot.

[36] Cox BJ, McWilliams LA, Enns MW, Clara IP (2004). Broad and specific personality dimensions associated with major depression in a nationally representative sample. Compr Psychiatry.

[37] Southwick SM, Yehuda R, Giller EL (1995). Psychological dimensions of depression in borderline personality disorder. Am J Psychiatry.

[38] Sharhabani-Arzy R, Amir M, Swisa A (2005). Self-criticism, dependency and posttraumatic stress disorder among a female group of help-seeking victims of domestic violence in Israel. Pers Individ Differ.

[39] Cox BJ, MacPherson PS, Enns MW, McWilliams LA (2004). Neuroticism and self-criticism associated with posttraumatic stress disorder in a nationally representative sample. Behav Res Ther.

[40] Kelly AC, Carter JC (2013). Why self-critical patients present with more severe eating disorder pathology: the mediating role of shame. Br J Clin Psychol.

[41] Cox BJ, Fleet C, Stein MB (2004). Self-criticism and social phobia in the US national comorbidity survey. J Affect Disord.

[42] Fazaa N, Page S (2009). Personality style and impulsivity as determinants of suicidal subgroups. Arch Suicide Res.

[43] Klomek AB, Orbach I, Sher L, Sommerfeld E, Diller R, Apter A (2008). Quality of depression among suicidal inpatient youth. Arch Suicide Res.

[44] Frost RO, Marten P, Lahart C, Rosenblate R (1990). The dimensions of perfectionism. Cogn Ther Res.

[45] Hewitt PL, Flett GL (1991). Perfectionism in the self and social contexts: conceptualization, assessment, and association with psychopathology. J Pers Soc Psychol.

[46] Gilbert P, Clarke M, Hempel S, Miles JN, Irons C (2004). Criticizing and reassuring oneself: an exploration of forms, styles and reasons in female students. Br J Clin Psychol.

[47] Blatt SJ, Zuroff DC (1992). Interpersonal relatedness and self-definition: two prototypes for depression. Clin Psychol Rev.

[48] Curran T, Hill A (2019). Perfectionism is increasing over time: a meta-analysis of birth cohort differences from 1989 to 2016. Psychol Bull.

